# Enhanced Stability and Performance of α-FAPbI_3_ Photodetectors via Long-Chain n-Heptanoic Acid Passivation

**DOI:** 10.3390/ma19010122

**Published:** 2025-12-30

**Authors:** Xintao Bai, Yunjie Lou, Mengxuan Wang, Zhenkun Gu, Yanlin Song

**Affiliations:** 1 Henan Institute of Advanced Technology, Zhengzhou University, Zhengzhou 450001, China; baixintao1227@163.com (X.B.); louyunjie0502@163.com (Y.L.); wangmengxuan1129@163.com (M.W.); 2 Institute of Chemistry, Chinese Academy of Sciences, Beijing 100190, China; 3 University of Chinese Academy of Sciences, Beijing 100049, China

**Keywords:** α-FAPbI_3_ photodetectors, broad-spectrum and high-sensitivity detection, stability

## Abstract

Owing to its narrow bandgap and excellent thermal stability, formamidinium–lead triiodide (FAPbI_3_) is a promising perovskite for high-performance, wide-spectrum photodetectors. Here, we selected long-chain n-heptanoic acid as the passivating agent and introduced it onto the perovskite surface via post-treatment, thereby enabling the fabrication of high-quality α-FAPbI_3_ perovskite films and photodetectors. It is found that the carboxylic acid group in the n-heptanoic acid molecule can effectively passivate crystal defects, greatly reduce the density of defect states in the perovskite film, and inhibit the non-radiative recombination of carriers. The α-FAPbI_3_ perovskite phase was effectively stabilized. The responsivity of the photodetector optimized by n-heptanoic acid is as high as 0.47 A W^−1^ at 740 nm. At the same time, the optimized device still maintains 95% of its initial performance after 552 h of storage in an air environment with a room temperature of 25 °C and a relative humidity of 25%. This method provides a reliable way to prepare a high-performance and stable α-FAPbI_3_ photodetector.

## 1. Introduction

In recent years, perovskite materials have emerged as one of the most promising candidates for photovoltaic applications, owing to their exceptional optoelectronic properties, including high quantum yield, high absorption coefficient, high carrier mobility, long carrier diffusion length, and tunable bandgap [1,2,3,4,5]. These attributes have facilitated their widespread application in various optoelectronic devices, such as perovskite solar cells [6,7,8], photodetectors [9,10,11], light-emitting diodes [12,13,14], and lasers [15,16,17]. As research on perovskite materials advances, perovskite-based photodetectors have also become a focal point of current investigations, demonstrating immense research potential and promising application prospects. Notably, perovskite photodetectors offer the advantages of low fabrication costs, as the perovskite layer can be synthesized via solution-based methods at relatively low temperatures, enabling the production of flexible devices. In terms of performance, perovskite photodetectors exhibit a broad responsive spectrum (spanning from ultraviolet–visible to near-infrared light), rapid response speeds, high sensitivity under low-light conditions, low noise current, and robust detection capabilities [18,19,20,21,22]. The rapid advancement of perovskite photodetectors is inextricably linked to the research on perovskite thin-film materials, as the light-absorbing layer of perovskite significantly influences the device performance and stability of perovskite photodetectors. Among various perovskite materials, α-FAPbI_3_ perovskite holds significant promise for wide-spectrum photodetector applications, owing to its narrow bandgap and excellent thermal stability [23,24,25]. Therefore, the fabrication of high-quality, pure formamidinium-based perovskite films with enhanced stability at room temperature is of paramount importance for effectively improving the performance and stability of α-FAPbI_3_-based perovskite photodetectors, thereby facilitating their industrial-scale production and application.

Currently, a large number of studies focus on incorporating materials containing the −COOH group into perovskites to reduce defects in perovskite films and enhance crystallinity. Compared to short-chain carboxylic acids, long-chain carboxylic acids show better hydrophobicity due to their larger nonpolar surface areas. Their extended carbon skeletons also enable them to readily form ordered aggregates via intermolecular van der Waals forces, and the extended conjugation along the chains allows for a broader electron delocalization range [26]. Therefore, long-chain carboxylic acids are increasingly being utilized by researchers as passivating agents. Sun et al. introduced 4,4′-sulfonyldibenzoic acid (SA) into perovskite precursor solutions. The −C=O in −COOH coordinated with Pb^2+^ to slow film growth, while −OH formed hydrogen bonds with I^−^, passivating iodine-related defects. The SA-modified photodetector exhibited a peak external quantum efficiency of 89.88%, an ultra-low dark current density of 1.46 × 10^−10^ A cm^−2^, and notably improved stability [27]. Fu et al. doped lead pyridine-2-carboxylate, featuring −COOH groups, into perovskite, regulating crystallization and passivating grain boundaries to yield high-quality films with larger grains and fewer defects [28]. The introduction of −COOH can reduce defects in perovskite films and enhance crystallinity to obtain high-quality perovskite films to improve device performance.

Herein, long-chain n-heptanoic acid was selected as a passivating agent and introduced onto the perovskite surface through a post-treatment process, enabling the fabrication of high-quality α-FAPbI_3_ perovskite films and photodetectors. The results indicate that the carboxylic acid group in n-heptanoic acid effectively passivates crystal defects, substantially reducing the defect-state density within the perovskite film and suppressing non-radiative carrier recombination, thereby stabilizing the α-FAPbI_3_ phase. Benefiting from this improved film quality, the n-heptanoic-acid-optimized photodetector achieves a responsivity of 0.47 A·W^−1^ at 740 nm. Moreover, the optimized device retains 95% of its initial performance after 552 h of storage under ambient conditions (25 °C and 25% relative humidity). This approach provides a novel and effective strategy for the preparation of high-performance and stable α-FAPbI_3_ photodetectors.

## 2. Materials and Methods

### 2.1. Materials

The patterned indium tin oxide (ITO) substrates and 2,2′,7,7′-tetrakis (N, N-dip-methoxyphenylamine)-9,9′-spirobifluorene (Spiro-OMeTAD, 99.86%) were purchased from Advanced Election Technology Corp, Dalian, China. N-Heptanoic acid (C_7_H_14_O_2_; 98%) was purchased from Macklin Biochemical Technology Co., Ltd, Shanghai, China. The SnO_2_ colloid precursor (15 wt.%) and chlorobenzene (spectrophotometric grade, 99.8%) were obtained from Alfa Aesar, Shanghai, China. Lead iodine (PbI_2_), formamidine iodine (FAI), lithium bis (trifluoromethanesulfonyl) imide (Li-TFSI), 4-t-butylphenylammonium iodide (tBP), and 3,5-difluorobenzylamine (97%) were purchased from Xi’an Yurisolar Technology Co., Ltd., Xi’an, China. N, N-dimethylformamide (DMF, 99.9%), dimethyl sulfoxide (DMSO, 99.9%), and thiourea were purchased from Sigma-Aldrich, Shanghai, China. All these materials were used as received without further purification.

### 2.2. Device Fabrication

The ITO glasses were ultrasonically cleaned with detergent, deionized water, acetone, and ethanol for 20 min in turn. The N_2_ blow-dried ITO substrates were treated with 15 min ultraviolet ozone. Then the diluted SnO_2_ dispersion (2.14 wt.%) was spun onto the ITO substrate at 3000 rpm for 30 s, and the as-prepared film was annealed at 180 °C for 20 min. After cooling to room temperature, the SnO_2_/ITO substrates were transferred into the N_2_ glove box. 1.6 M FAPbI_3_ was dissolved in a mixed solvent of DMF and NMP (6:1 by volume ratio). The perovskite film was prepared by a one-step spin-coating procedure at 5000 rpm for 30 s. Then, 300 μL chlorobenzene antisolvent was added dropwise after 17 s from the start of spin coating and then placed on a heating platform at 100 °C for annealing for 1 min and 150 °C for annealing for 15 min. At this stage, the perovskite film acts as the control sample. For the experimental sample, a chlorobenzene solution with 0.3 mg/mL n-heptanoic acid molecules is spin-coated onto the control. The spin coater runs at 5000 rpm for 30 s, with the vacuum pump on and the hot plate set to 150 °C. Using a 100 µL pipette, evenly apply the solution onto an ITO substrate coated with an SnO_2_ layer and a black perovskite film. After spinning stops, quickly transfer the substrate to the 150 °C hot plate, anneal for 5 min, and then set it aside for later use. For the hole transport layer (HTL) solution, the Spiro-OMeTAD solution was prepared by mixing 72.3 mg Spiro-OMeTAD, 17.5 μL Li-TFSI solution (520 mg Li-TFSI in 1 mL acetonitrile), and 29 μL tBP in 1 mL chlorobenzene, which was spin-coated on top of the perovskite layer at 3000 rpm for 30 s. Finally, 100 nm Ag was thermally evaporated as an electrode using a shadow mask (0.04 cm^2^).

### 2.3. Characterization

UV–vis absorption spectra were measured by a Cary 5000 spectrophotometer (Agilent Technologies, Santa Clara, CA, USA). The photoluminescence (PL) spectra and the time-resolved PL (TRPL) spectra were obtained using a fluorescence spectrometer (HORIBA FluoroMax, Kyoto, Japan). The X-ray diffraction (XRD) patterns were recorded on the Bruker X-ray diffractometer (Rigaku Corporation, Tokyo, Japan) using Cu Kα. The top and cross-sectional morphology of reference and n-heptanoic acid molecule-modified films were measured by field-emission scanning electron microscopy (SEM, EDAX Octane Supper, FEI Nova NaoSEM, ZEISS-Sigma 300, Oberkochen, Germany). The X-ray photoelectron spectroscopy (XPS) data were obtained by an X-ray photoelectron spectrometer (Thermo Scientific ESCALAB Xi^+^, Waltham, MA, USA). The external quantum efficiency (EQE), responsivity, and detectivity were measured using a semiconductor characterization system (PD-QE, ENLITECH, Taiwan, China). The response times were obtained from PD-RS, ENLITECH. Space charge limiting current (SCLC) was tested by the solar battery carrier characteristic analyzer (PAIOS of Fluxim, Schwyz, Switzerland). The electrochemical impedance spectroscopy (EIS), transient photocurrent (TPC), transient photovoltage (TPV), and Mott–Schottky plots were measured by a solar battery carrier characteristic analyzer (PAIOS of Fluxim). The proton nuclear magnetic resonance (^1^H NMR) spectrum was measured using a BRUKER AVANCE III HD 400 spectrometer (Bruker, Billerica, MA, USA). The stability test was conducted by placing the samples in a constant-temperature and constant-humidity cabinet at 25 °C and 25% RH under room temperature conditions.

## 3. Results and Discussion

**Figure 1:**
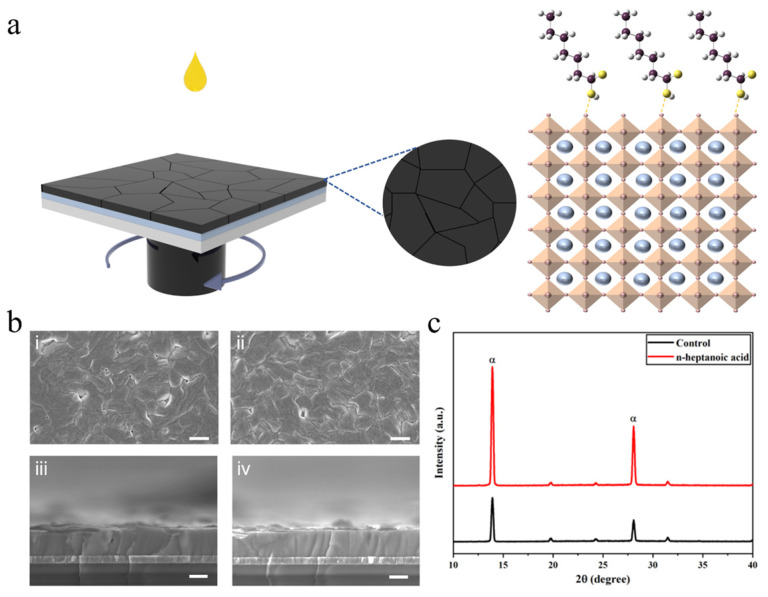
(**a**) Schematic diagram of the post-treatment of the perovskite film with n-heptanoic acid molecules. (**b**) SEM top-view images of (**i**) the control film and (**ii**) the film with n-heptanoic acid molecules, scale bar: 2 µm. SEM cross-sectional images of (**iii**) the control film and (**iv**) the film with n-heptanoic acid molecules, scale bar: 0.5 µm. (**c**) XRD patterns of the control and n-heptanoic acid films.

Figure 1a presents the schematic diagram of the post-treatment of the perovskite film with n-heptanoic acid molecules. Specifically, after the perovskite film is prepared by the one-step spin-coating method and has completed annealing, a chlorobenzene solution containing 0.3 mg/mL n-heptanoic acid molecules is spin-coated onto the perovskite film. We characterized the control and n-heptanoic acid-treated perovskite film morphology using scanning electron microscopy (SEM) (Figure 1b). As clearly observed from the images, the control perovskite film exhibited a significant number of void defects. In contrast, the perovskite films treated with n-heptanoic acid demonstrated a marked reduction in void defects, more uniform grain size, and vertically penetrating crystals, along with a slight increase in the grain size of the perovskite crystals. Three-dimensional profilometer tests showed that the surface roughness of the control group perovskite film is 26.06 nm, while the n-heptanoic acid molecules-regulated film’s roughness is reduced to 16.62 nm, consistent with the SEM results (Figure S1). To investigate the impact of n-heptanoic acid on the crystallinity of perovskite films, we carried out X-ray diffraction (XRD) measurements (Figure 1c). Compared with the control perovskite film, the α-FAPbI_3_ perovskite film treated with n-heptanoic acid molecules exhibited significantly increased peak intensities at the (110) and (220) planes. The results indicate that the crystallinity of the film was markedly enhanced after n-heptanoic acid treatment [29].

**Figure 2:**
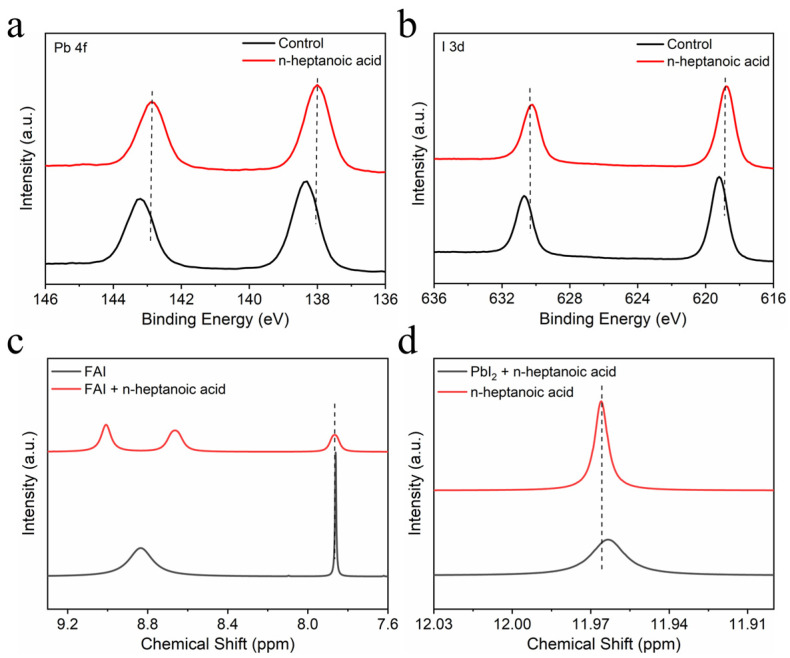
The XPS spectra of (**a**) Pb 4f orbital and (**b**) I 3d orbital for the control and n-heptanoic acid film samples. (**c**) ^1^HNMR spectra of FAI and FAI + n-heptanoic acid. (**d**) ^1^H NMR spectra of n-heptanoic acid and n-heptanoic acid + PbI_2_.

To probe the interaction between n-heptanoic acid molecules and perovskite, we prepared both control perovskite films and those treated with n-heptanoic acid, followed by X-ray photoelectron spectroscopy (XPS) measurements to examine changes in the binding energy of uncoordinated Pb^2+^ (Figure 2a,b). The peaks related to Pb 4f in the n-heptanoic acid-treated perovskite films shifted toward lower binding energies, indicating a strong interaction between the carboxyl groups in the n-heptanoic acid passivation molecules and the perovskite. Furthermore, the I 3d spectra of the n-heptanoic acid-treated perovskite films also exhibited a shift in the peak position towards a smaller binding energy, implying an interaction between n-heptanoic acid molecules and the Pb-I octahedra. To verify the interaction between n-heptanoic acid and perovskite, we conducted nuclear magnetic resonance hydrogen spectroscopy (^1^H-NMR) tests. As illustrated in Figure 2c, when n-heptanoic acid interacts with FAI, the proton peak of the -NH_2_ group in FAI shifts, and the peak of C=NH_2_^+^ splits [30]. This indicates the formation of stable hydrogen bonds between n-heptanoic acid and FAI. As shown in Figure 2d, the position of the −COOH peak shifts, suggesting the existence of a coordination interaction between n-heptanoic acid and Pb^2+^.

**Figure 3:**
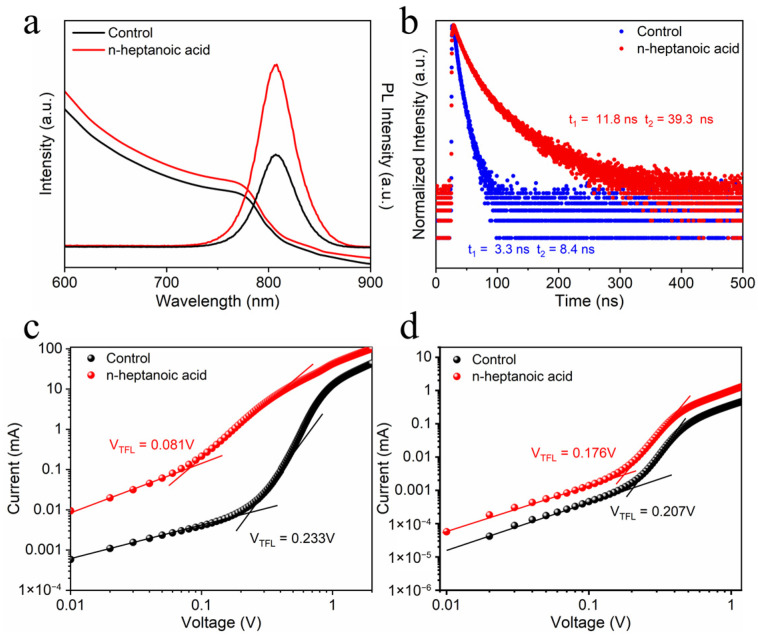
(**a**) Steady-state PL spectra and UV-vis absorption of perovskite films on quartz substrates. (**b**) The TRPL spectra of the control and n-heptanoic acid perovskite films. Space charge limited current (SCLC) measurements for perovskite films modified with n-heptanoic acid in devices with (**c**) a pure electron transport layer and (**d**) a pure hole transport layer.

To substantiate the photoluminescence (PL) properties of n-heptanoic acid, we selected n-caproic acid and n-butanoic acid with different chain lengths (Figure S2). Under identical conditions, we measured their photoluminescence spectra. The results revealed that the perovskite films treated with n-heptanoic acid exhibited the highest PL intensity. Subsequently, we measured their steady-state photoluminescence (PL) and ultraviolet-visible (UV-Vis) spectra to evaluate the impact of n-heptanoic acid molecules on the quality of perovskite films (Figure 3a). The post-treated film with n-heptanoic acid molecules showed higher absorption across 600–900 nm, indicating improved crystallinity. Compared to the control perovskite film, the PL emission peak of the perovskite film after n-heptanoic acid post-treatment was significantly enhanced, suggesting that the carboxyl groups could passivate defects and suppress non-radiative recombination in the perovskite. This aligns with the dark-condition electrochemical impedance spectroscopy (EIS) findings, showing higher composite impedance in the n-heptanoic acid device than in the control, thus confirming enhanced suppression of non-radiative recombination (Figure S3). Figure 3b exhibits the TRPL spectra of the control and n-heptanoic acid perovskite films (the fitting formula and results can be found in Table S1, Supplementary Materials). The two decay components τ_1_ and τ_2_ are attributed to non-radiative recombination from defects, and radiative recombination from the bulk perovskite, respectively [31,32]. The average carrier lifetimes (τ_ave_) of the control and n-heptanoic acid perovskite films are 3.3 and 16.3 ns, respectively, indicating that the presence of the n-heptanoic acid can reduce the non-radiative carrier recombination, suppress the defect formation in the perovskite, and improve the film quality of the perovskite.

To compare the trap density (N_trap_) of the control and n-heptanoic acid perovskite films, a space-charge-limited current (SCLC) was tested (Figure 3c,d). The structure of the device adopts an electron-only structure of ITO/SnO_2_/Perovskite/n-heptanoic acid/PCBM/Ag and a hole-only structure of ITO/PEDOT/Perovskite/n-heptanoic acid/Spiro-OMeTAD/Ag, respectively. The trap-filled limit voltage (V_TFL_) can be obtained by fitting the mutational point of SCLC curves, that is, the demarcation point of the ohmic region and trap-filled region. Accordingly, the V_TFL_ has a relationship with the N_trap_ satisfying the Equation [33]:

(1) $$V_{TFL}=\frac{eL^{2}N_{trap}}{2\varepsilon_{0}\varepsilon }$$

where e is the elementary charge (e = 1.60 × 10^−19^ C), L is the film thickness (700 nm), ε_0_ is the vacuum permittivity (ε_0_ = 8.854 × 10^−12^ F m^−1^), and ε is the relative dielectric constant of perovskite (ε = 32). In the SCLC measurements conducted on devices with a pure electron transport layer structure, the V_TFL_ was 0.233 V, and the N_trap_ was 1.68 × 10^14^ cm^−3^ for the control film, whereas it was 0.081 V and the N_trap_ was 5.85 × 10^13^ cm^−3^ for the n-heptanoic acid perovskite film. In the SCLC tests on devices with a pure hole transport layer structure, the V_TFL_ was 0.207 V, and the N_trap_ was 1.50 × 10^14^ cm^−3^ for the control film and 0.176 V the N_trap_ was 1.27 × 10^14^ cm^−3^ for the n-heptanoic acid perovskite film. These results indicate that the defect density in the n-heptanoic acid perovskite film is lower than that in the control film, suggesting that n-heptanoic acid molecules effectively passivate defects at the grain boundaries and within the bulk phase of the perovskite. This also corroborates the TRPL results, indicating that the corresponding photoluminescence lifetime increases as a consequence of the reduced defect density.

**Figure 4:**
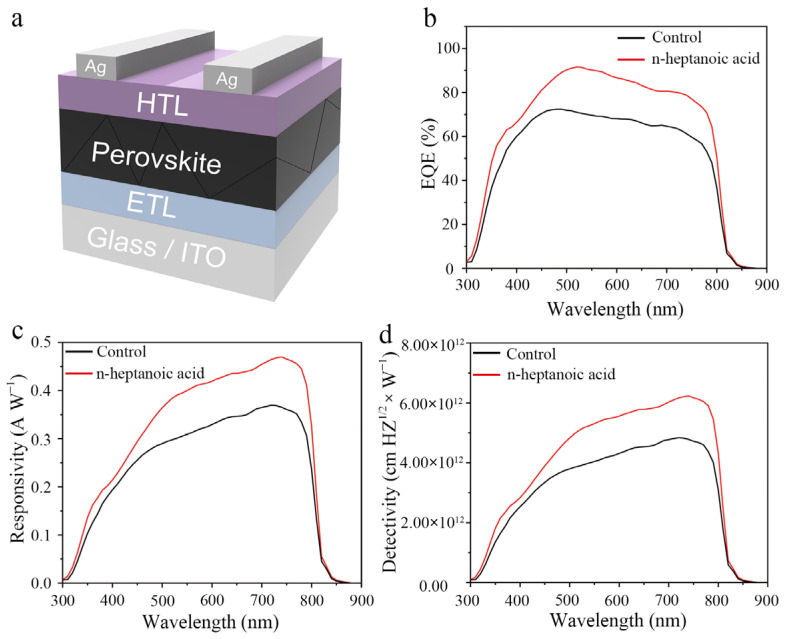
(**a**) The schematic device structure of perovskite photodetectors. (**b**) The EQE of the control and n-heptanoic acid device under different wavelengths of incident light. (**c**) The R of the control and n-heptanoic acid device under different wavelengths of incident light. (**d**) The detectivity of the control and n-heptanoic acid device under different wavelengths of incident light.

We fabricated a vertically structured n–i–p-type perovskite photodetector with the configuration ITO/SnO_2_/FAPbI_3_/Spiro-OMeTAD/Ag and systematically characterized its optoelectronic performance. The device architecture is illustrated in Figure 4a. As shown in Figure 4b, the maximum external quantum efficiency (EQE) of the device fabricated with the control perovskite film is 72% at 480 nm under zero-bias conditions. For the device treated with n-heptanoic acid, the EQE exceeds 85% across the wavelength range of 470 to 620 nm, suggesting improved photoelectric conversion efficiency. We measured the transient photocurrent and transient photovoltage of the devices (Figures S4 and S5). The results revealed that the performance of the n-heptanoic acid devices surpassed that of the control devices, indicating that n-heptanoic acid molecules can effectively suppress exciton recombination induced by defects, thereby enhancing both the stability and photoelectric performance of the devices. The responsivity (R) represents the efficiency of a device in responding to optical signals is calculated using the following relation [34]:

(2) $$R=\frac{EQE\times q\times \lambda }{hc}$$

where q is the elementary charge, λ is the wavelength, *h* is Planck’s constant, and c is the speed of light. Through calculation, it was found that within the wavelength range of 300 nm to 900 nm, the control sample achieves a maximum responsivity (R) of 0.37 A W^−1^ at 740 nm. In contrast, the photodetector device treated with n-heptanoic acid reaches a peak of 0.47 A W^−1^ at 740 nm, which represents an improvement of 0.10 A W^−1^ and enables broad-spectrum detection (Figure 4c). Under zero external bias, the optimized device demonstrates an exceptional R value, indicating its capability as a self-powered perovskite photodetector that efficiently converts optical signals into electrical signals.

We tested the device’s optical switch response under a 405 nm wavelength laser and 0 V bias voltage. Compared to the control device, the target device exhibited a higher optoelectronic voltage switching ratio (Figure S6). After obtaining the curves depicting the variation in the responsivity (R) with wavelength and the dark current for the photodetectors fabricated based on the control perovskite film and the perovskite film treated with n-heptanoic acid molecules, we calculated the corresponding detectivity using the following formula [35,36]:

(3) $$D^{*}\approx \frac{\sqrt{2}R}{\sqrt{2eI_{Dark}}}$$

R represents the responsivity at different wavelengths, e denotes the elementary charge, and I_dark_ is the dark current measured under zero-bias voltage. As shown in Figure 4d, the specific detectivity (D*) of the device fabricated from the film post-treated with n-heptanoic acid is generally higher than that of the original device within the wavelength range of 300–900 nm. The control device achieves a maximum D* value of 4.84 × 10^12^ cm Hz^1/2^ W^−1^ at 740 nm. In contrast, for the optimized device, it reaches a peak value of 6.23 × 10^12^ cm Hz^1/2^ W^−1^ at 740 nm. Since the n-heptanoic acid device has improved photoelectric performance, we speculate that it has a higher built-in potential (V_bi_). The Mott–Schottky plot (Figure S7) shows a V_bi_ of 1.08 V for the control device and 1.12 V for the n-heptanoic acid device. The improved V_bi_ significantly facilitates directional charge transport at the interface, which reduces charge accumulation and recombination, leading to an increase in V_OC_.

**Figure 5:**
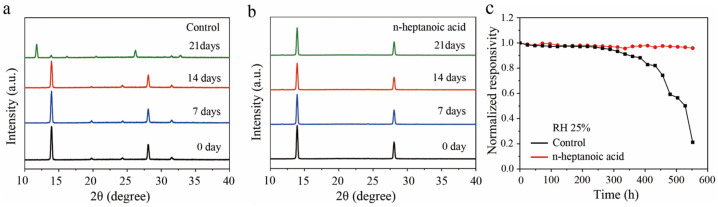
The XRD stability of (**a**) the control and (**b**) n-heptanoic acid-treated perovskite film during 21 days. (**c**) The normalized responsivity variation curves of the photodetectors with RH 25%.

Compared with the control perovskite films, the n-heptanoic acid-treated perovskite films show a nearly 17-degree increase in contact angle, indicating enhanced hydrophobicity that can consequently reduce the impact of moisture on the perovskite device (Figure S8). The phase stability of perovskite films was examined by recording XRD patterns over various aging periods under ambient conditions (25 °C, RH 25%) (Figure 5a,b). Compared with the control perovskite film, after 21 days of aging in air, the n-heptanoic acid-treated perovskite film exhibits no significant decomposition, demonstrating favorable stability. Subsequently, we evaluated the stability of the control and n-heptanoic acid photodetectors in air with a humidity of RH 25%. According to the normalized responsivity variation curves (Figure 5c), the humidity stability of the n-heptanoic acid device is superior to the control, maintaining 95% of its original responsivity after over 552 h, while the responsivity of the control device declines to nearly zero after the same time aging.

## 4. Conclusions

To address the phase instability issue in FAPbI_3_ perovskite, we propose a passivation strategy using n-heptanoic acid molecules. Employing a one-step solution method, we fabricated highly efficient and stable α-FAPbI_3_ photodetectors. The passivated films show enhanced PL intensity, prolonged carrier lifetimes, and reduced non-radiative recombination, with n-heptanoic acid effectively mitigating crystal defects. The carboxylic acid groups in n-heptanoic acid passivate uncoordinated Pb^2+^ ions, significantly improving device performance (responsivity up to 0.47 A W^−1^ at 740 nm). Moreover, the optimized devices retain 95% of their initial performance after 552 h in ambient conditions (25 °C, RH 25%). This approach offers a promising way to enhance α-FAPbI_3_ photodetector performance and stability.

## Data Availability

The original contributions presented in this study are included in the article/Supplementary Material. Further inquiries can be directed to the corresponding authors.

## Supplementary Materials

The following supporting information can be downloaded at https://www.mdpi.com/article/10.3390/ma19010122/s1, Figure S1: (a) Surface profilometry characterization of the perovskite film in the control group. (b) Surface profilometry characterization of the perovskite film treated with n-heptanoic acid molecules. Figure S2: Photoluminescence spectra of the perovskite films treated with carboxylic acids of different chain lengths. Figure S3: Impedance spectrum of the control and n-heptanoic acid devices in the dark. Figure S4: Transient photocurrent (TPC) results for the control and n-heptanoic acid devices. The TPC lifetime of the n-heptanoic acid-modified devices is 418 ns, which is shorter than that of the control device (534 ns). Figure S5: Transient photovoltage (TPV) results for the control and n-heptanoic acid devices. The TPV lifetime of the device functionalized with n-heptanoic acid is measured at 282.2 μs, which is notably longer than that of the control device (181.3 μs). Figure S6: Optical switching response curves of photodetectors prepared based on the control perovskite films and n-heptanoic acid molecules-modified perovskite films. Figure S7: Mott–Schottky plots of the control and n-heptanoic acid devices. Figure S8: (a) Contact angles of the perovskite films in the control group: left 57.4°, right 57.6°. (b) Contact angles of the perovskite films treated with n-heptanoic acid molecules: left 74.1°, right 74.6°. Table S1: Fitting parameters of the TRPL spectra for the control and n-heptanoic acid perovskite films.
