# Supplementary material for: Enhanced Stability and Performance of α-FAPbI3 Photodetectors via Long-Chain n-Heptanoic Acid Passivation
Source: Materials (Basel). 2025 Dec 30;19(1):122. doi: 10.3390/ma19010122 (PMC12787277; doi:10.3390/ma19010122)
Supplement: Supplementary file 1 [file materials-19-00122-s001.zip › materials-4056092-supplementary.pdf]

Supplementary Material

Enhanced Stability and Performance of α-$FAPbI_3$ Photodetectors via Long-Chain n-Heptanoic Acid Passivation

*Xintao Bai, Yunjie Lou, Mengxuan Wang, Zhenkun Gu *and Yanlin Song **

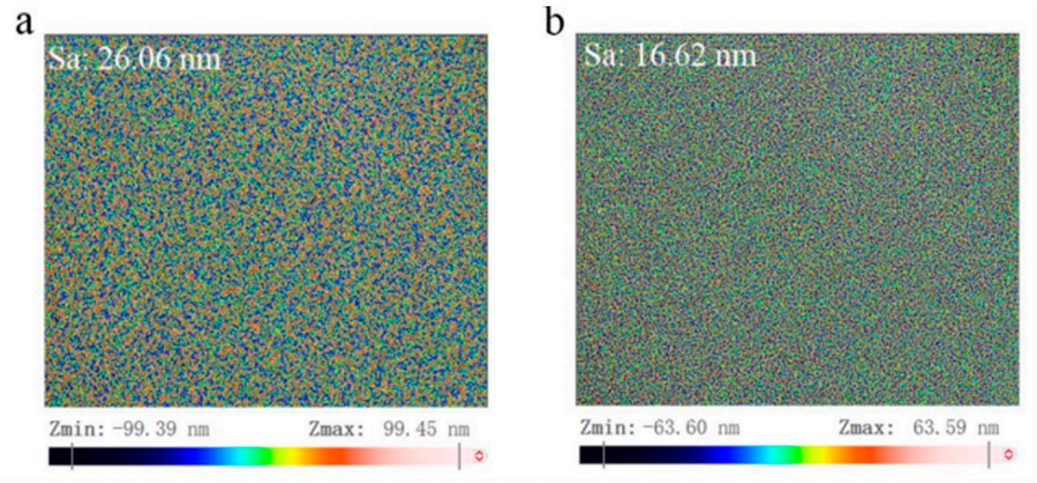

Figure S1. (a) Surface profilometry characterization of the perovskite film in the control group. (b) Surface profilometry characterization of the perovskite film treated with n-heptanoic acid molecules.

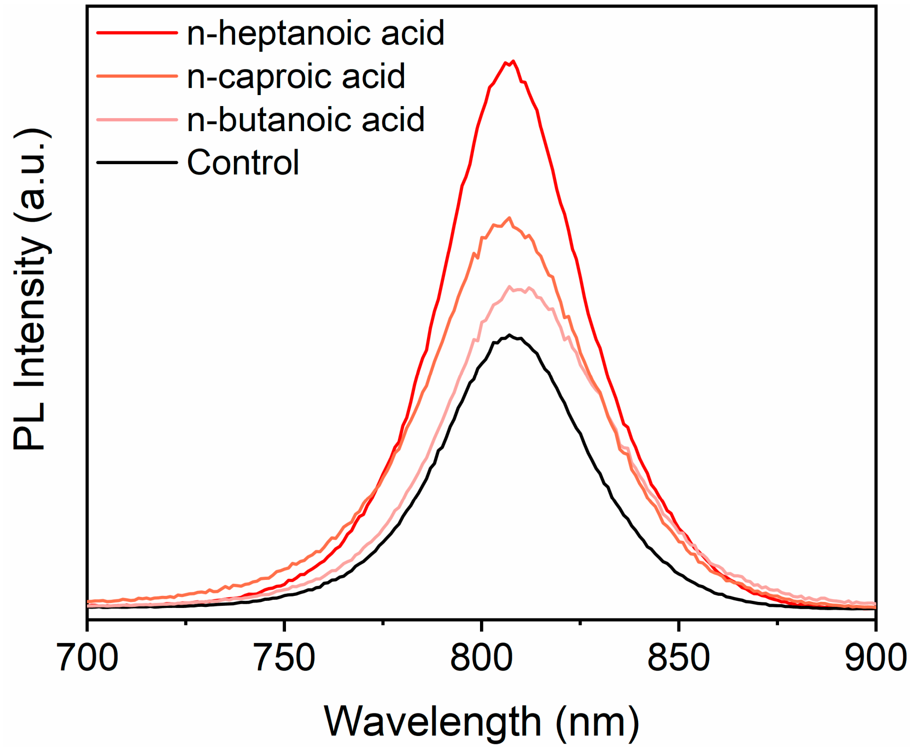

Figure S2. Photoluminescence spectra of the perovskite films treated with carboxylic acids with different chain lengths.

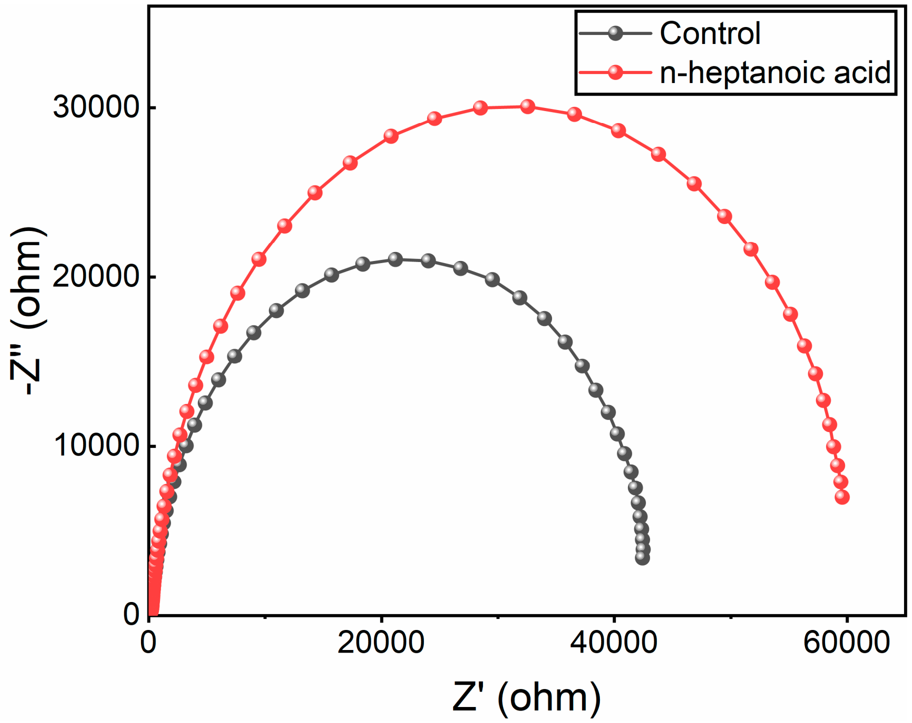

Figure S3. Impedance spectrum of the control and n-heptanoic acid devices in the dark.

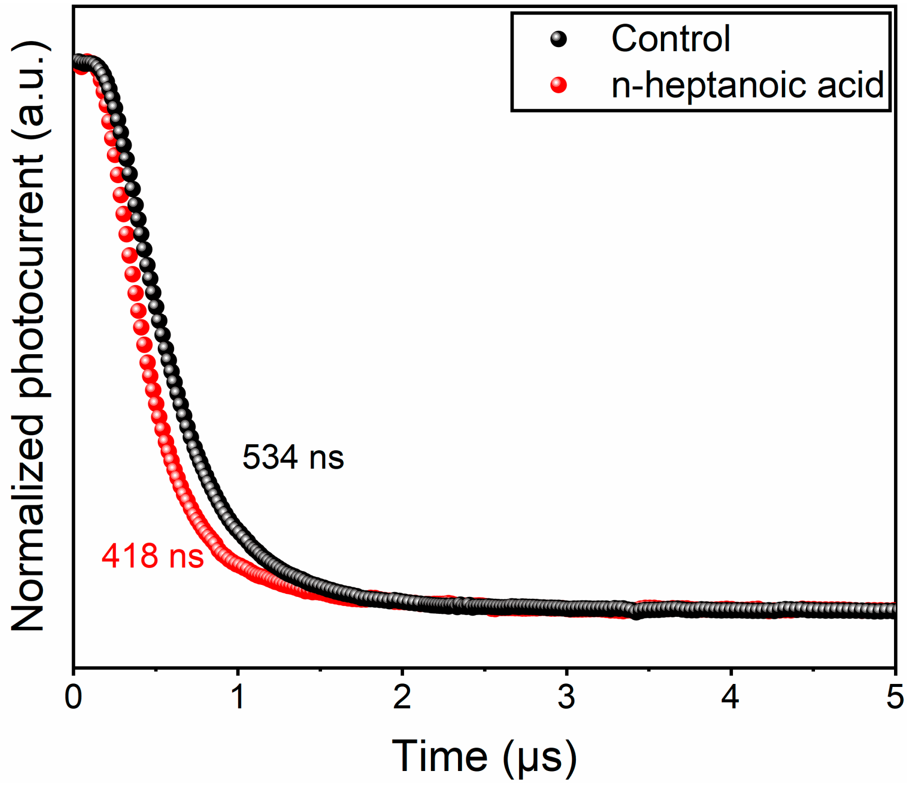

Figure S4. Transient photocurrent (TPC) results for the control and n-heptanoic acid devices. The TPC lifetime of the n-heptanoic acid-modified devices is 418 ns, which is shorter than that of the control device (534 ns).

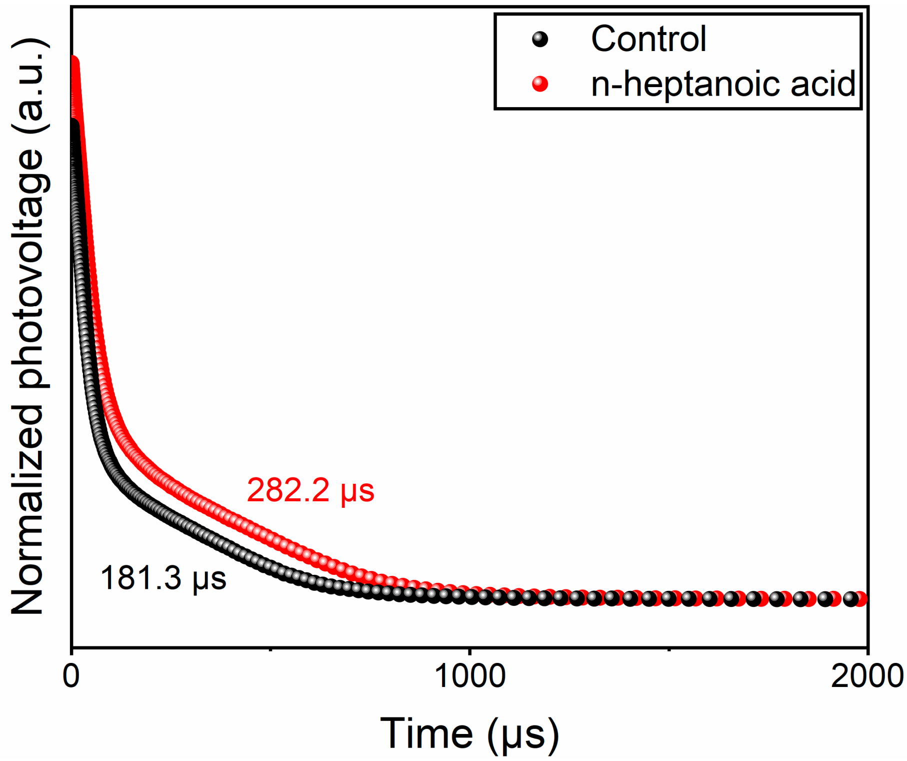

Figure S5. Transient photovoltage (TPV) results for the control and n-heptanoic acid devices. The TPV lifetime of the device functionalized with n-heptanoic acid is measured at 282.2 μs, which is notably longer than that of the control device (181.3 μs).

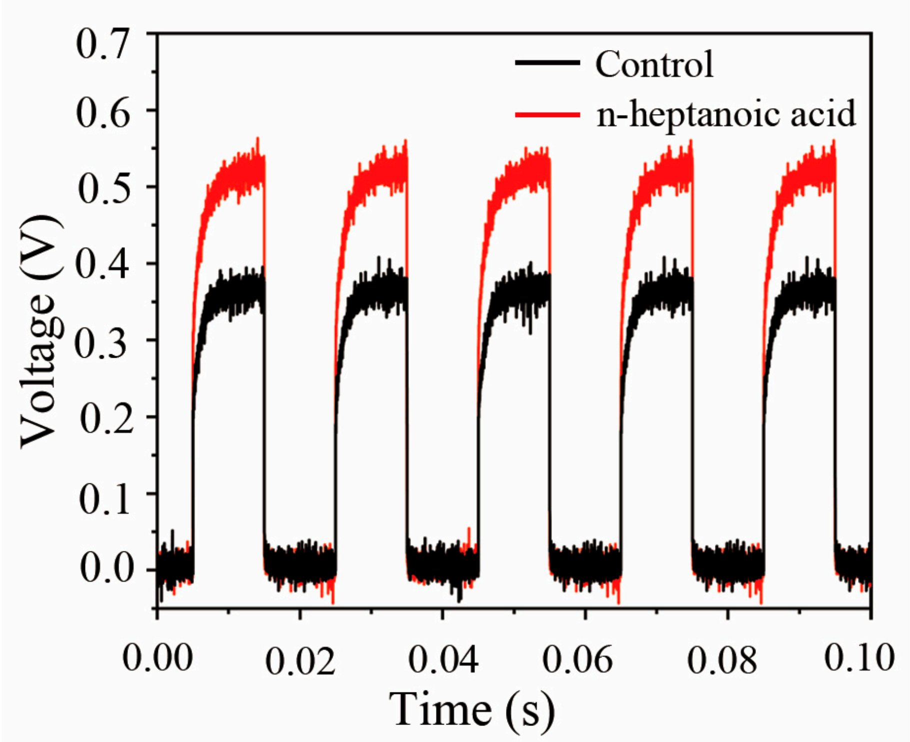

Figure S6. Optical switching response curves of photodetectors prepared based on the control perovskite films and n-heptanoic acid molecules-modified perovskite films.

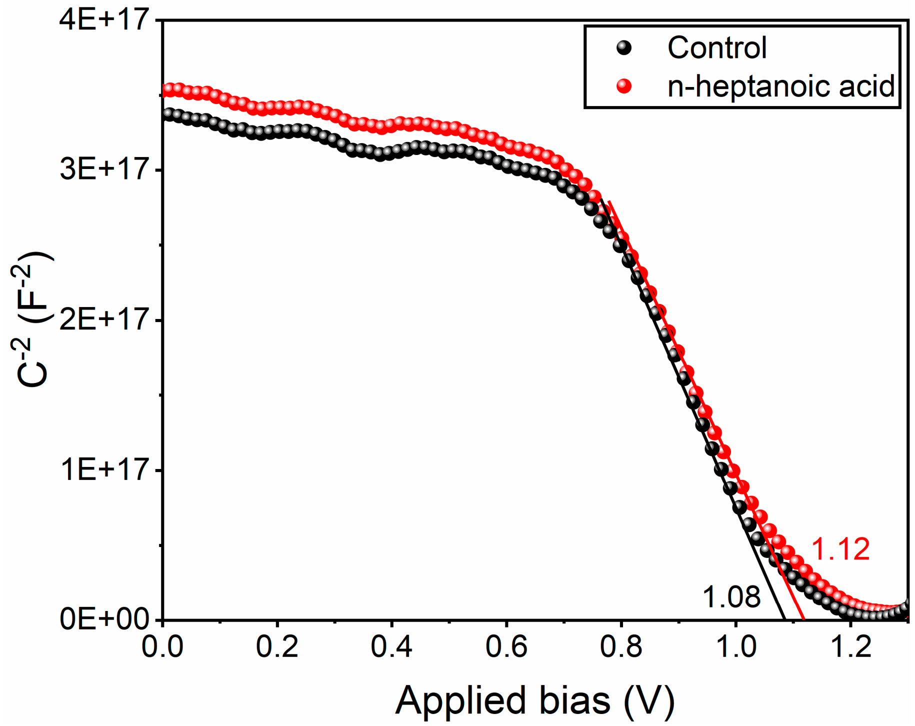

Figure S7. Mott-Schottky plots of the control and n-heptanoic acid devices.

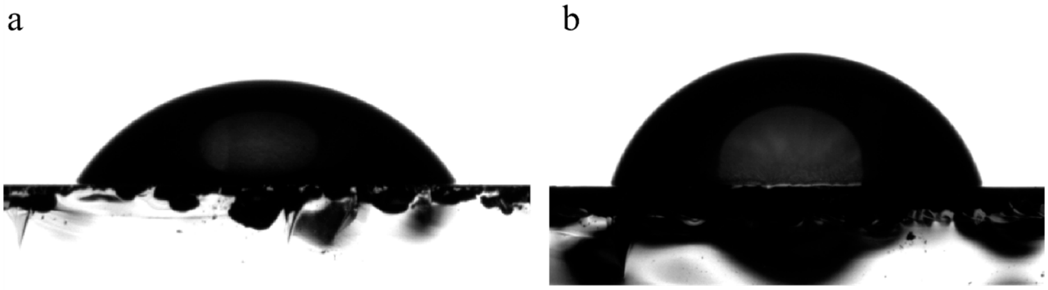

Figure S8. (a) Contact angles of the perovskite films in the control group: left 57.4°, right 57.6°. (b) Contact angles of the perovskite films treated with n-heptanoic acid molecules: left 74.1°, right 74.6°.

Table S1. Fitting parameters of the TRPL spectra for the control and n-heptanoic acid perovskite films.

	A_1(%)	τ_1(ns)	A_2(%)	τ_2(ns)
Control	99.73	3.3	0.27	8.4
n-heptanoic acid	94.27	11.8	5.73	39.3

PL decay fitting curve is based on the bi-exponential decay equation:

$$f(t) = A_1 \exp\left(\frac{-t}{\tau_1}\right) + A_2 \exp\left(\frac{-t}{\tau_2}\right) + y_0 \quad (1)$$

Where A_1 and A_2 represent the decay amplitude, τ_1 represents trap-assisted recombination, τ_2 represents free carrier recombination, and y_0 is a constant for baseline offset.
